# Narrow band ultraviolet B irradiations cause alteration in interleukin-31 serum level in psoriatic patients

**DOI:** 10.1007/s00403-012-1293-6

**Published:** 2012-10-30

**Authors:** Joanna Narbutt, Irmina Olejniczak, Dorota Sobolewska-Sztychny, Anna Sysa-Jedrzejowska, Iwona Słowik-Kwiatkowska, Tomasz Hawro, Aleksandra Lesiak

**Affiliations:** 1 Department of Dermatology, Medical University of Lodz, 5 Krzemieniecka, 94-017 Lodz, Poland; 2 Department of Internal Medicine, Endocrinology and Diabetology, Lublin Hospital, Lublin, Poland

**Keywords:** Narrow band UVB, Neuropeptides, Psoriasis vulgaris, IL-31

## Abstract

Scientific communications indicate the disturbed expression of neuropeptides in the skin and serum in psoriasis vulgaris (PsV) patients. Narrow-band ultraviolet radiation (NB-UVB) is one of the systemic therapies of PsV. The aim of the study was to evaluate the influence of NB-UVB therapy on substance P (SP), calcitonin gene-related peptide (CGRP), brain-derived neurotrophic factor (BDNF), corticotropin-releasing factor (CRF) and interleukin-31 (IL-31) serum concentrations in PsV patients. 59 psoriatic patients with mean PASI (psoriasis area and severity index) 14.3 were treated with NB-UVB (20 exposures). The control group consisted of 50 healthy subjects, whose age and sex matched. In all patients, serum concentration of BDNF, CRF, IL-31 substance P and CGRP was analyzed by ELISA before the treatment and in psoriatic group the analysis was also done after 10 and 20 irradiations. In patients there was found a significantly higher concentration of IL-31 (215.3 vs. 748.6 ng/ml; *p* < 0.0001), SP (25.7 vs. 67.2 pg/ml; *p* < 0.01), CGRP (31.4 vs. 44.15 pg/ml; *p* < 0.01) and a lower concentration of CRF (0.89 vs. 0.426 ng/ml; *p* < 0.0001) and BDNF (16.39 vs. 14.15 ng/ml; *p* = 0.1216) in comparison with the controls. 20 NB-UVB exposures caused a significant decrease in IL-31 level (748.6 vs. 631.7 ng/ml; *p* < 0.0001). The NB-UVB therapy had no major effect on neuropeptides serum levels regardless of a number of irradiations. On the basis of our study it can be suggested that IL-31 is involved in pathogenesis of psoriasis and the NB-UVB therapy causes alterations in its level.

## Introduction

Psoriasis is one of the most common skin diseases, affecting 1.5–3 % population. It is a chronic, inflammatory, non-infectious disorder, whose pathogenesis is still not fully elucidated. Genetic and immunologic factors, including the increased proliferation of keratinocytes under the influence of activated T cells, the effects of proinflammatory cytokines, as well as autoimmune factors should be accounted [6].

The ability of neuropeptides to initiate cutaneous inflammation is crucial to our understanding of their putative role in psoriasis [20].

There are various neuropeptides involved in inflammatory skin disorders, which include substance P (SP), calcitonin gene-related peptide (CGRP), brain-derived neurotrophic factor (BDNF), corticotropin-releasing factor (CRF) and interleukin 31 (IL-31) [20, 25].

Many scientific communications indicate the disturbed expression of neuropeptides in skin and serum in patients suffering from psoriasis vulgaris (PsV) [8]. Farber et al. [8] suggested that neuropeptides presented in unmyelinated dermal nerve endings may be of pathogenic importance in psoriasis. Chapman et al. [4] and Halevy et al. [9] reported that activation of the autonomic nervous system causes an increase in the expression of selected neuropeptides and, in consequence, it leads to the development of psoriatic lesions. The importance of the nervous system in psoriasis is supported by the observation that skin lesions resolve in places of sensory denervation [17, 18].

One of the very important symptoms of psoriasis is pruritus. Almost 80 % of patients have a moderate to severe pruritus and many of them have generalized itching. Despite being common symptom in psoriasis, the pathogenesis of pruritus is far to be well understood.

Recent studies have demonstrated that pruritus may be caused by local inflammation and impairment in neuropeptides expression [23, 28]. We examined IL-31 in psoriatic patients because itch is one of the clinical features of the disease; however, its mechanism is still unclear. IL-31 plays a role in pathogenesis of itch, thus we decided to check its level in psoriatic patients [18, 19].

Many scientific communications indicate that the intensity of psoriatic pruritus correlates with the degree of quality of life impairment and the state of depression [18, 23, 27]. Reich et al. [18] suggest that the presence and intensity of pruritus in psoriatic patients may be related to perceived stress.

One of the most common systemic therapies of PsV is the treatment with a narrow-band ultraviolet irradiation (NB-UVB). Due to biological properties of UVB, skin exposure to ultraviolet results in erythema, inflammation and immunosuppression [11]. Only a few studies have analysed the influence of NB-UVB irradiation on the neuropeptides serum concentration in psoriatic patients. Thus, the objective of this study was to evaluate the influence of NB-UVB therapy on SP, CGRP, BDNF, CRF and IL-31 serum concentrations in PsV patients.

## Materials and methods

The study group included 59 patients with PsV treated with NB-UVB therapy at the Department of Dermatology and Venereology, Medical University of Lodz between 2007 and 2009. The mean age was 47 years old (29–63 years old) and the mean duration of the disease was 20 years (from 6 months to 40 years). 56 healthy non-stressed controls (age and sex matched) were enrolled into the study. All individuals gave written informed consent before entering the study. The experimental plan was approved by the local ethics committee of the Medical University of Lodz and was conducted according to the principles of the Declaration of Helsinki. Before treatment, the intensity of psoriatic lesions was assessed in all patients by PASI. The mean PASI index before phototherapy was 14.3. In addition, the clinical assessment of itch was done with the use of 20-points scale based on Reich’s pruritus questionnaire severity [18, 19].

The patients had II and III skin phototype as assessed by Fitzpatrick scale. The patients underwent irradiations with UVB of wavelength 311–312 nm for 20 consecutive days with initial dose of 0.7 personal MED. They were treated in a Dermalight–Medisun 2800 PC-AB cabin (Schulze and Böhm GmbH–Brühl, Germany) with TL100W/01 fluorescent lamps (Philips, Eindhoven, Netherlands). The mean initial dose was 0.2 J/cm^2^ and it was systematically increased either daily or every other day, depending on the individual patient’s reaction. The mean cumulative dose of UVB 311 radiation was 12.7 J/cm^2^. This regimen is used at our Department for several years and treatment results are satisfactory. The same doses were used also in other studies [1].

Before treatment, a 7.5-ml blood sample was taken to measure the concentration of CRF (Yanaihara Institute INC. Shizuoka, Japan), BDNF (Raybiotech INC. Norccross, USA) and IL-31 (R&D System, Minneapolis Abingdon, USA) with the use of ELISA. Additionally, in 24 patients SP (R&D System, Abingdon, UK) and CGRP (Bertin Pharma, Montigny le Bretonneux, France) concentration was also determined. These procedures were repeated after the 10th and 20th irradiation. To avoid circadian variation in neuropeptides level, blood was drawn only at morning hours i.e., 8–9 am both from psoriatic patients and control group. The obtained results were statistically analysed.

The statistical analysis was performed using Kruskal–Wallis and Dunn’s Multiple Comparison Test. The test was statistically significant if *p* < 0.05.

## Results

Serum concentrations of all analysed proteins in all subjects are presented in Tables 1, 2 and 3.

**Table 1:** IL-31, CRF, BDNF, SP and CGRP serum level in healthy subjects and psoriatic patients

	Median serum level–control group	Median serum level–psoriatic group	*p* value
IL-31 (ng/ml)	215.3	748.6	**<0.0001**
CRF (ng/ml)	0.89	0.426	**<0.0001**
BDNF (ng/ml)	16.39	14.15	>0.05
SP (pg/ml)	25.7	67.2	**<0.01**
CGRP (pg/ml)	31.4	44.15	**<0.01**

Bold values are statistically significant (*p* < 0.05)

**Table 2:** IL-31, CRF, BDNF serum level in psoriatic patients before during and after NB-UVB therapy

	IL-31			CRF			BDNF		
	Baseline	10 UVB	20 UVB	Baseline	10 UVB	20 UVB	Baseline	10 UVB	20 UVB
Number of subjets	59	59	59	59	59	59	59	59	59
Minimum	22.33	89.88	57.76	0.17	0.194	0.186	1.549	6.146	4.277
25 % Percentile	608	363.5	438.6	0.334	0.321	0.363	9.789	8.777	10.72
Median level ng/ml	748.6	1112	631.7	0.426	0.426	0.449	14.15	12.82	14.15
75 % Percentile	3,153	1,920	1,595	0.7	0.69	0.705	19.29	20.76	18.68
Maximum	4,853	4,419	6,009	1.173	1.145	1.08	72.94	71.75	72.7
Mean level (ng/ml)	1,669	1,429	1,123	0.5011	0.5099	0.5156	16.69	16.9	16.03

**Table 3:** SP and CGRP serum level in psoriatic patients before during and after NB-UVB therapy

	SP			CGRP		
	Baseline	10 UVB	20 UVB	Baseline	10 UVB	20 UVB
Number of subjects	24	24	24	24	24	24
Minimum	4.0	0.5	0.5	30.7	26	26
25 % Percentile	33.65	24.9	22.75	35.5	31.9	32
Median level pg/ml	67.2	46.9	53.35	44.15	39.6	37.9
75 % Percentile	101.5	75.3	91.1	57.4	47.95	45.4
Maximum	677.3	153.4	278.4	121.1	92.5	80
Mean level (pg/ml)	102.6	53.23	69.3	52.05	44.28	43.24

**Fig. 1:**
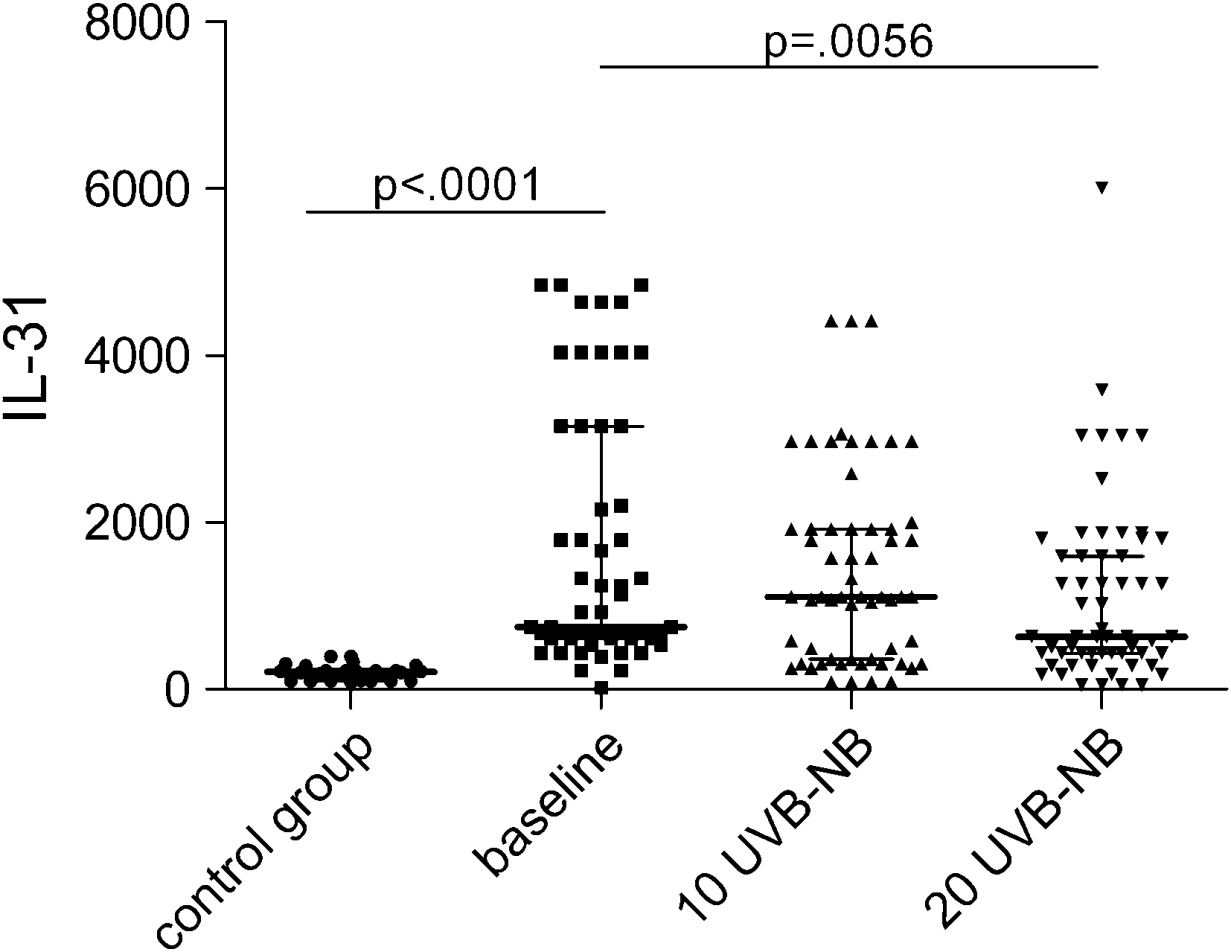
Changes of IL-31 serum concentration during phototherapy

In analysed groups, a significantly higher concentration of IL-31 was observed among psoriatic patients in comparison with the control group (748.6 vs. 215.3 ng/ml; *p* < 0.0001). After first 10 NB-UVB irradiations, concentration of IL-31 raised indifferently (*p* > 0.05) while next ten exposures provoked its significant decrease to 631.7 ng/ml (*p* < 0.01) (Fig. 1).

The CRF serum level was significantly lower in psoriatic patients than that in the control group (0.426 vs. 0.89 ng/ml; *p* < 0.0001). Irradiation with NB-UVB (both 10 and 20 exposures) did not provoke any changes in CRF concentration (0.426 ng/ml, 0.449 ng/ml, respectively; *p* = 0.1376).

The BDNF plasma level was not different for patients and healthy subjects (14.15 vs. 16.39 ng/ml; *p* = 0.1216) and NB-UVB irradiations caused no changes in this parameter (12.82 ng/ml after 10 treatments and 14.15 ng/ml after 20 treatments; *p* = 0.5350).

In patients with PsV, SP serum concentration was significantly higher than that in controls (67.2 vs. 25.7 pg/ml; *p* < 0.01). Under NB-UVB indifferent changes in SP level were observed (46.9 pg/ml after 10 and 53.35 pg/ml after 20 irradiations; *p* > 0.05 for both comparisons). The baseline CGRP concentration was significantly higher among psoriatic patients in comparison with the control group (44.15 vs. 31.4 pg/ml; *p* < 0.01). The concentration of CGRP during phototherapy did not change statistically (39.6 pg/ml after 10 exposures and 37.9 pg/ml after 20 doses; *p* > 0.05 for both comparisons).

PASI score improved significantly from 14.3 to 3.5 after 20 irradiations (*p* < 0.05). PASI dropped to 4.0 after 10 irradiations and to 3.5 after additional 10 irradiations. The lack of significant improvement in PASI index from 10th to 20th irradiations may testify for photoadaptation.

In the examined group 80 % (*n* = 47) of patients suffered from pruritus. During phototherapy the sensation of pruritus decreased gradually. Mean pruritus score was seven before therapy and decreased significantly to 2.4 after 10 irradiations and to 1.05 after 20 irradiations (*p* < 0.05 for both comparisons). Mean PASI before treatment was significantly higher in the group with pruritus (22.2) when compared to the patients without itch (12.1; *p* < 0.05). 40 out of 59 patients showed no symptoms of itch after phototherapy (grade = 0). There were no correlations between PASI index and itch score or between PASI index or itch score and IL-31, SP, CGRP, CRF and BDNF levels (*p* > 0.05 for all comparisons).

## Discussion

Recent data indicate psoriasis as systemic disease, which may lead to impairment of the quality of life, and pruritus is believed to be a key phenomenon involved in this process. Thus, neuropeptides involved in itch pathogenesis are widely studied nowadays. IL-31 is a newly described cytokine produced by Th2 lymphocytes. It causes a biological effect by binding with a heterodimeric receptor consisted of two subunits: IL31RA and OSMR. The receptor is expressed on epithelial cells and keratinocytes. IL-31 stimulation induce an activity of various chemokines, indicating that IL-31 takes part in the recruitment of polymorphonuclear cells, monocytes and T cells to sites of skin inflammation in vivo [7].

The overexpression of IL-31 in transgenic mice caused alopecia and chronic pruritus, which led to development of skin lesions similar to those observed in atopic dermatitis.

Numerous studies indicate that IL-31 may play a role in development of many dermatoses i.e., atopic dermatitis, allergic contact dermatitis, chronic urticaria and prurigo nodularis [8].

Single researches have demonstrated elevated levels of IL-31 in the skin of patients with PsV [2]. Most studies concerned mRNA expression of IL-31 and its receptor. To our knowledge, serum level of this cytokine in psoriatic patients has not been evaluated yet. Our research results show that the concentration of IL-31 in the analyzed group was significantly higher than that in healthy individuals, which confirms its significance in pathogenesis of psoriasis. In addition, the decrease of its concentration during irradiations, accompanied by the decrease of pruritus, is a sign that IL-31 may play the role in pathogenesis of psoriatic pruritus [19, 22, 24].

CRF is a central component of a hypothalamic–pituitary–adrenal axis and it is an important coordinator of the systemic stress response. In peripheral sites, a cutaneous CRF and a CRF-receptor1 is believed to regulate various skin functions that are important for local homeostasis [5, 26, 29].

Numerous researches focused on the role of CRF in patients with psoriasis. Other authors [27] indicated that psoriatic patients showed markedly low CRF values in serum, which confirms our results. In another paper, Zhou et al. [29] evaluated CRF and CRF-R1 expression in psoriatic lesions by immunohistochemistry and they found a lower expression of CRF and CRF-R1 in psoriatic lesions in comparison with normal control skin. This observation is coherent with the data obtained by Tagen et al. [25].

However, there are no consistent results on this subject as in other studies, O’Kane et al. [13], as well as Kim et al. [10], reported that a CRF protein expression increased in the affected skin of active psoriatic patients in comparison with the control. On the base of our study, we can observe a significantly lower serum concentration of CRF in psoriatic patients when compared with the control group. Some results of CRF expression in psoriasis are conflicting so it is necessary to confirm by further studies that a CRF system is aberrant in psoriatic skin lesions.

A brain-derived neurotrophic factor is a member of a nerve growth factor family. In fact, BDNF can be found in a range of tissues and cell types, and recently its pivotal role has been described in allergic inflammation. The significance of this neurotrophin in psoriasis still remains unknown [15]. Most recent studies have concentrated on the role of BDNF in pathogenesis of atopic dermatitis. Raap et al. [16] analysed circulating levels of brain-derived neurotrophic factor in AD, which they compared with both patients with psoriasis and nonatopic healthy subjects. They did not find increased level of BDNF in patients with psoriasis, which is consistent with the results of our study.

Within a group of 24 patients we additionally analysed the correlation between the SP and CGRP serum level and the intensity of pruritus.

In our study, the concentration of SP and CGRP in the control group was significantly lower when compared to the patients with psoriasis. No changes in SP serum level were observed in pruritic versus non-pruritic psoriatic patients (data not shown). Our results may suggest that SP is not a direct pruritus indicator and the higher concentration is caused by local inflammation [18].

We observed the opposite situation in case of CGRP, which was significantly higher in the group without pruritus. Reich et al. [18] noted that overexpressed neuropeptides in psoriatic lesions in the group with pruritus would lead to a higher expression and activity of proteases and it may contribute to the decrease in their concentration [14, 21].

In 2007, Reich et al. [8] published a study describing a higher level of SP and a lower CGRP level in a group of patients without pruritus. Nonetheless, Nakamura et al. [12] did not notice an increase in expression of CGRP in psoriatic skin from patients with pruritus in comparison with non-pruritic individuals.

While evaluating the influence of NB-UVB irradiations on the SP serum concentration, we showed only minimal changes in level of this neuropeptide. In addition, these alterations were similar in groups with and without pruritus. The new research should be conducted to confirm these results in a larger group of patients.

Although we did not assess the correlation between psoriasis severity and serum level of SP and CGRP, their decrease during therapy can be linked with an immunosuppressive action of phototherapy or may be secondary to remission of psoriatic changes. This suggestion was confirmed by Reich et al. [18].

Within the examined group we found a higher PASI in pruritic patients, which was also established by Szepietowski et al. [23] and Chang et al. [3]. No correlation between the pruritus intensity and the psoriasis activity (assessed by PASI) was pointed by Nakamura et al. [12]. These divergent results require further explanation.

There is no doubt, as our observations show that there is an influence of UVB 311 irradiations on pruritus sensation. During the therapy, pruritus was systematically reduced and in two-thirds of patients it totally receded. The suppression of pruritus indicator, such as IL-31, is a possible explanation for our findings. However, it cannot be ultimately determined that these results are secondary to the remission of psoriatic changes and skin inflammation.
